# Sequence type 8 as an emerging clone of methicillin-resistant *Staphylococcus aureus* causing bloodstream infections in Taiwan

**DOI:** 10.1080/22221751.2021.1981158

**Published:** 2021-09-24

**Authors:** Pao-Yu Chen, Yu-Chung Chuang, Jann-Tay Wang, Wang-Huei Sheng, Yee-Chun Chen, Shan-Chwen Chang

**Affiliations:** a Department of Internal Medicine, National Taiwan University Hospital, Taipei, Taiwan; b Graduate Institute of Clinical Medicine, College of Medicine, National Taiwan University, Taipei, Taiwan; c National Institutes of Infectious Diseases and Vaccinology, National Health Research Institutes, Zhunan, Taiwan; d College of Medicine, National Taiwan University, Taipei, Taiwan

**Keywords:** USA300, multilocus sequence typing, spa typing, PVL, ACME

## Abstract

Sequence type (ST) 8 has not been a common methicillin-resistant *Staphylococcus aureus* (MRSA) clone in Asia until recently. We aimed to determine the clinical significance and microbiological characteristics of MRSA bacteraemia (MRSAB) caused by ST8 and other endemic clones. A total of 281 non-duplicated MRSAB were identified in a medical centre between 2016 and 2018. Sequencing of target genes was performed to determine ST and to confirm ST8 belonging to USA300. Antimicrobial susceptibility testing was performing by using Sensititre standard panel. In total, ST8 accounted for 18.5% of MRSAB ranking after ST239 (31.0%) and ST59 (23.5%). However, it increased to become the most prevalent clone finally. All ST8 isolates belonged to *spa* clonal complex008, and carried SCC*mec* IV/IVa, PVL and ACME genes, indicating USA300. ST8/USA300 isolates were highly susceptible to non-β-lactams antibiotics, except fluoroquinolone and erythromycin. ST8/USA300 MRSAB is commonly developed in community settings with either healthcare risks or not (71.2%). Compared to other STs MRSAB, ST8/USA300 MRSAB patients had more diabetes mellitus (50.0%), more admitted from long-term care facility residents (25.0%), had more skin ad soft tissue infection as primary focus (25.0%), and had fewer vascular devices (26.9%) at MRSAB onset. On multivariable analysis, isolates with vancomycin MIC were significantly associated with mortality in the dose–response relationship, rather than STs. This report depicts the clinical features of ST8/USA300 MRSAB and clonal shift from prior endemic clones to ST8/USA300. Our data strongly support long-term surveillance to ascertain whether ST8/USA300 will successfully disseminate and demonstrate its pathogenicity on clinical outcomes.

## Introduction

Methicillin-resistant *Staphylococcus aureus* (MRSA) is one of the important drug-resistant pathogens and causes high disease burdens globally [1,2]. Efforts into infection control and implementation of antibiotic stewardship have seen the secular trends of the incidence of hospital-acquired (HA)-MRSA infections decrease, but community-acquired (CA)-MRSA infections are still prevalent worldwide, including Taiwan [3,4].

Sequence type (ST) 8 has been the dominant clone causing CA-MRSA infections in both North and South America for two decades, while genotypes of CA-MRSA in Asia are diverse and country-specific. For example, ST59 was dominant in Taiwan and China, ST72 in South Korea, ST5 in Japan, and ST30 in southeast Asia, while ST80 is prevalent in the background of genetic heterogeneity in Europe [5,6]. Though clone differences of CA-MRSA around the world have been characterized, the predominant clinical presentations of these CA-MRSA clones are skin and soft tissue infections [5,6]. Additionally, these clones commonly carry staphylococcal cassette chromosome (SCC) *mec* types IV or V without antibiotic-resistant genes, except *mec*A [5,6]. This genetic feature consequently leads to CA-MRSA being less resistant to non-β-lactams.

Clonal expansion of CA-MRSA clones and its subsequent introduction to the hospital environment were observed in the past decades. As intermixing of CA- and HA-MRSA clones, several specific CA-MRSA clones further replaced the original HA-MRSA clones to become prevalent in both communities and hospitals [3–6]. In the United States (USA), USA100 (ST5), the traditional HA-MRSA clone has been substituted by USA300 (ST8) [4–6], defined as possessing similar banding patterns by pulsed-field gel electrophoresis (PFGE) and having the following characteristics: *spa*-clonal complex (CC)008, methicillin resistance, Panton-Valentine Leukocidin (PVL) and Arginine Catabolic Mobile Element (ACME)-encoding genes [7,8]. The dominance of ST8 is less reported in continents other than the USA, but ST8 has not uncommonly been identified in clinical specimens in Asia since 2010 [9,10]. Prior studies from Japan and South Korea demonstrated that ST8 spreads in both communities and hospitals to become an emerging clone causing MRSA infections, further confirming that ST8 belongs to USA300 under the aforementioned definitions [9,10].

In Taiwan, ST59 used to be the major CA-MRSA clone, while the proportions of ST59 in HA-MRSA bacteraemia (HA-MRSAB) increased as high as that of the HA-MRSA clone ST239 after 2010 [11]. Additionally, ST45 has emerged since 2010 with the highest carriage rates and environmental contamination of ST45 in long-term care facilities [12,13]. In parallel, one longitudinal multicentre study between 1995 and 2015 suggested ST8 is also emerging among MRSA colonization and infection isolates by showing most ST8 (85%) identified after 2010 [14]. A later study demonstrated ST8, in second place after ST59, accounted for 20% of the collected MRSA isolates causing cellulitis or osteomyelitis between 2016 and 2018 [15]. The findings that ST8 have abruptly increased in non-bloodstream invasive infections raises the question as to whether ST8 subsequently has become prevalent and replaced prior endemic MRSA clones causing bacteraemia in Taiwan. In addition, it is important to ascertain whether ST8 MRSA in Taiwan was genetically related to USA300.

In this study, we planned to test clonal shift to ST8 in the contemporary collection of MRSA bloodstream isolates, to infer the genetic relationship of ST8 and USA300, and further to compare clinical characteristics, antibiotic susceptibility testing and patient outcomes among ST8 and other STs in Taiwan.

## Materials and methods

### Patients

From 1 January 2016 to 31 December 2018, all adult patients age ≥20 years with MRSAB admitted to National Taiwan University Hospital (NTUH), a major tertiary teaching hospital with 2500 beds located in northern Taiwan, were retrospectively enrolled in this study. MRSAB was defined as ≥1 culture from a blood sample yielding MRSA with compatible symptoms/signs [16]. If a patient had multiple episodes of MRSAB during the study period, only the first episode was considered in the present study. This study was approved by the Institutional Review Board (IRB) at NTUH (NTUH-201701082RIND, NTUH-202102038RINA).

### Definitions

A standardized case report form was used to collect the patients’ demographic, clinical, and routine laboratory data, including the patient’s age and sex, the primary foci of the bacteraemia, defined by the U.S. Centers for Disease Control and Prevention [17], presence of shock within 24 h of the onset of MRSAB, the underlying diseases and conditions, the Charlson co-morbidity index, and Pitt bacteraemia score at the time of onset of MRSAB. We epidemiologically categorized patients with MRSAB into three groups: hospital-acquired (HA; index culture obtained ≥48 h after admission); healthcare-associated, community onset (HACO; at least one healthcare-associated risk factor); and CA (community-associated). Healthcare-associated risk factors included hospitalization within one year before MRSAB, residence in a long-term care facility for any duration, surgery, dialysis, or presence of a central vascular catheter [16].

### Microbiological methods

The MRSA blood isolates have been prospectively preserved in the research laboratory of the Department of Internal Medicine at NTUH for more than 40 years. All preserved and available bacterial isolates were re-identified and confirmed as *S. aureus* by Gram stain, catalase-activity tests, and coagulase latex agglutination tests. MRSA was identified using CHROMagarTM MRSA plates. *In vitro* susceptibilities to erythromycin, clindamycin, gentamicin, oxacillin, tetracycline, trimethoprim/sulfamethoxazole (SXT), rifampin, ciprofloxacin, vancomycin, linezolid, and daptomycin were determined by the broth microdilution method using the Sensititre standard panel GPALL1F. The susceptibility test results were interpreted using the criteria provided by the Clinical Laboratory Standards Institute [18].

Genomic DNA was extracted using a Blood and Tissue Genomic DNA Miniprep system kit (VIOGENE) according to the instructions of the manufacturer. Multilocus sequence typing (MLST) was performed as described previously [19]. In brief, the *S. aureus* MLST scheme consisted of the seven housekeeping genes, including *arcC*, *aroE*, *glp*, *gmk*, *pta*, *tpi*, and *yqiL*. Fragments of each gene were used for PCR amplification, followed by sequencing. To assign allele numbers and sequence types, the sequence results of each locus were compared to the *S. aureus* MLST database (https://pubmlst.org/). A combination of the allele numbers generated from the seven gene fragments yields unique STs, and new allele numbers and STs numbers would be submitted to the database. The reference strain FPR3757, belonging to USA300 with multidrug resistance, was used as a standard for confirming whether a clinical MRSA isolate belonged to USA300. For those identified to be ST8 isolates, the presence of the SCC*mec* elements IV with subtyping IVa to IVd was determined by methods described previously [16,20], as well as the presence of PVL and ACME-encoding genes [16,21]. Analysis of the polymorphic X-region of the protein A gene (*spa*) and assignment of *spa* types were performed using the *Spa* typing plug-in tool of the BioNumerics software package (Applied Maths, Ghent, Belgium) [16]. Analysis of PFGE patterns was performed by using BioNumerics software (Applied Maths, Ghent, Belgium) [16]. Band position tolerance and optimization were set at 1.25 and 0.5%, respectively. Banding patterns within 80% similarity were considered the same pulsotype [7].

### Statistics

Continuous variables were described as medians and interquartile, and compared by using the Kruskal–Wallis one-way analysis of variance (ANOVA) with the Mann–Whitney *U* test as the post-hoc analysis. Categorical variables were described as percentages and compared by using the chi-square test with Bonferroni-adjusted α for pair-wise comparisons as post-hoc analysis. The time trend of rates of ST8 was examined by a logistic regression analysis. The predictors for all-cause in-hospital mortality were identified using logistic regression models. All parameters were initially tested using univariable analysis, and those with *p* values of <0.10 or biologically relevant variables were considered in the multivariable analysis. A stepwise model comparison and Akaike’s information criterion were used to determine the best model for the analysis of the multiple variables. All statistical analyses were performed using Stata software (version 14; StataCorp, College Station, TX). Two-sided *P* values less than 0.05 were considered significant.

## Results

**Figure 1.**
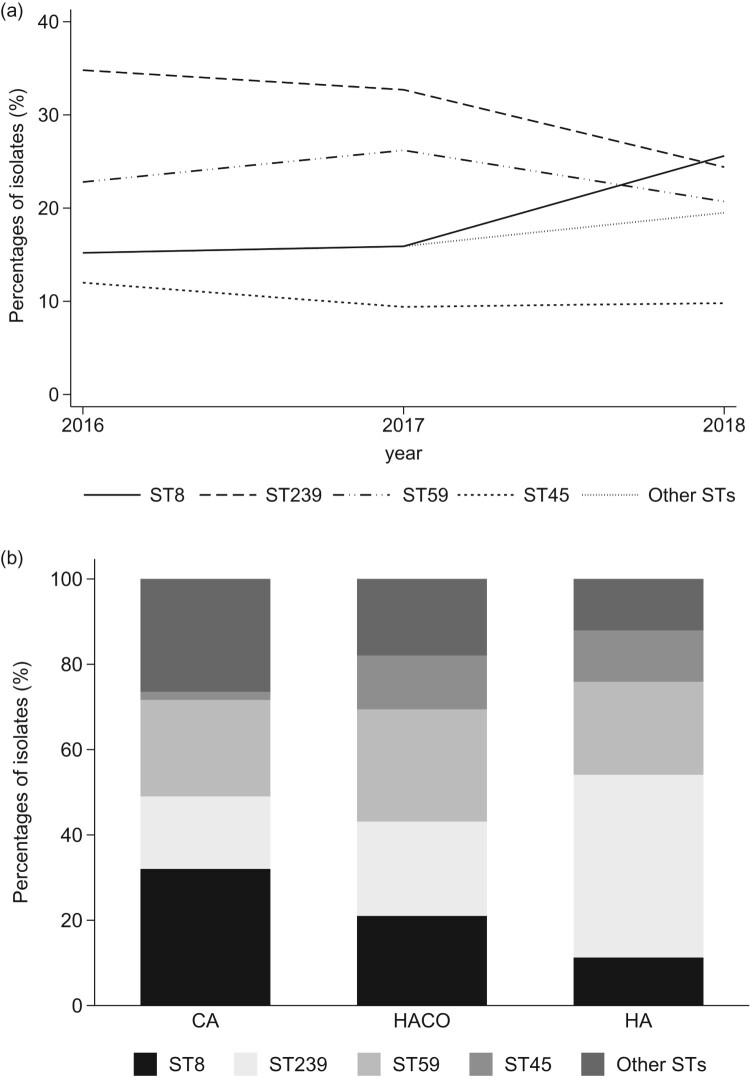
The change in the proportion of ST8, ST239, ST59, ST45, and other STs among 281 methicillin-resistant *Staphylococcus aureus* blood isolates by years (a) and by onset setting (b). Abbreviations are as follows: ST, sequence type; CA, community associated; HACO, healthcare-associated, community onset; HA, hospital acquired.

A total of 281 patients were found to have their first episode of MRSAB during 2016–2018. Overall, ST239 (87, 31.0%) and ST59 (66, 23.5%) remained the most common two sequencing types (Table 1). ST8 initially accounted for 15% and later jumped to 25% (*P* for trend, 0.08), paralleling the declines of both ST239 and ST59 in 2018. ST8 finally became the most prevalent ST (Figure 1(A); Supplementary Table 1). Most ST8 MRSAB developed in the community (71.2%, consisting of 17 CA-MRSAB and 20 HACO-MRSAB). The distribution of STs among the three onset settings was quite unique (Figure 1(B)). ST8 was the most common ST in CA-MRSAB, instead of ST59. Meanwhile, ST239 remained dominant in HA-MRSAB, and ST59 was equally distributed in three onset settings. ST45 was nearly all found in HACO-MRSAB and HA-MRSAB.

**Table 1.** Comparisons of clinical characteristics and outcomes of 281 patients with methicillin-resistant *Staphylococcus aureus* bacteraemia by sequencing types.

	Total (*n* = 281)	ST8 (*n* = 52)	ST239 (*n* = 87)	ST59 (*n* = 66)	ST45 (*n* = 29)	Other STs^a^ (*n* = 47)	*P-*value^b^				
							Overall	ST8 vs. ST239	ST8 vs. ST59	ST8 vs. ST45	ST8 vs. Other STs
**Demographics**											
Age, y, median (IQR)	68 (58–81)	75 (61.5–83.5)	66 (56–83)	68.5 (60–79)	68 (60–79)	67 (51–77)	0.09				
Gender, Male	178 (63.4)	34 (65.4)	61 (70.1)	41 (62.1)	18 (62.1)	24 (51.1)	0.30				
**Underlying diseases or conditions**											
Cardiovascular diseases	93 (33.1)	16 (30.8)	24 (27.6)	28 (42.4)	10 (34.5)	16 (31.9)	0.41				
Respiratory diseases	57 (20.3)	12 (23.1)	24 (27.6)	11 (16.7)	5 (17.2)	5 (10.6)	0.17				
Neurology diseases	51 (18.2)	14 (26.9)	15 (17.2)	6 (9.1)	4 (13.8)	12 (25.5)	0.07				
Hepatobiliary diseases	57 (20.3)	9 (17.3)	16 (18.4)	13 (19.7)	5 (17.2)	14 (29.8)	0.51				
Chronic renal impairment	91 (32.4)	17 (32.7)	30 (34.5)	20 (30.0)	9 (31.0)	15 (31.9)	0.99				
Chronic dialysis	44 (15.7)	8 (15.4)	14 (16.1)	9 (13.6)	5 (17.2)	8 (17.0)	0.99				
Rheumatology diseases	21 (7.5)	3 (5.8)	8 (9.2)	5 (7.6)	1 (3.5)	4 (8.5)	0.90				
Diabetes mellitus	92 (32.7)	26 (50.0)	23 (26.4)	18 (27.3)	9 (31.0)	16 (34.0)	**0**.**05**	**0**.**04**	0.09	0.80	0.90
Solid tumour	110 (39.2)	16 (30.8)	30 (34.5)	36 (54.6)	16 (55.2)	12 (25.3)	**0**.**003**^h^	>0.99	0.08	0.29	>0.99
Hematological malignancy	14 (5.0)	0 (0)	9 (10.3)	3 (4.6)	1 (3.5)	1 (2.1)	0.07				
Charlson comorbidity index, median (IQR)	4 (2–6)	4 (2–6)	4 (2–6)	5 (3–7)	4 (2–6)	4 (2–6)	0.32				
Long-term care facility	33 (11.7)	13 (25.0)	9 (10.3)	4 (6.1)	4 (13.8)	3 (6.4)	**0**.**02**	0.09	**0**.**02**	>0.99	**0**.**04**
Vascular device at onset^c^	137 (48.8)	14 (26.9)	61 (70.1)	30 (45.5)	14 (48.3)	18 (48.8)	<0.001	**<0**.**001**	0.38	0.55	>0.99
**Severity**											
Shock	75 (26.7)	15 (28.9)	33 (37.9)	12 (18.2)	6 (20.7)	9 (19.2)	**0**.**04**^i^	>0.99	>0.99	>0.99	>0.99
Pitt bacteremia score, median (IQR)	1 (0–3)	1 (0–3)	2 (1–5)	1 (0–2)	1 (0–3)	1 (0–3)	**0**.**02**^j^	0.84	>0.99	>0.99	>0.99
**Infection focus**											
Primary	105 (37.4)	24 (46.2)	25 (28.7)	24 (36.4)	12 (41.4)	20 (42.6)	0.26				
Catheter-related	71 (25.3)	5 (9.6)	32 (36.8)	15 (22.7)	10 (34.5)	9 (19.2)	**0**.**003**	**0**.**003**	0.98	0.12	>0.99
Device-related^d^	10 (3.4)	2 (3.9)	1 (1.2)	3 (4.6)	0 (0)	4 (8.5)	0.21				
Skin and soft tissue	49 (17.4)	13 (25.0)	12 (13.8)	9 (13.6)	3 (10.3)	12 (25.5)	0.17				
Pleuropulmonary^e^	39 (13.9)	6 (11.5)	18 (20.7)	6 (9.1)	2 (6.9)	7 (14.9)	0.23				
Native osteoarticular	7 (2.5)	1 (1.9)	2 (2.3)	3 (4.6)	1 (3.45)	0 (0)	0.64				
Endocarditis	14 (5.0)	4 (7.7)	5 (5.8)	3 (4.6)	1 (3.5)	1 (2.1)	0.82				
Septic thrombophlebitis	6 (2.1)	1 (1.9)	2 (2.3)	2 (3.0)	1 (3.5)	0 (0)	0.85				
Deep infection^f^	10 (3.6)	4 (7.7)	1 (1.2)	4 (6.1)	0 (0)	1 (2.1)	0.17				
**Management**											
Effective antibiotics within 48 h after onset	197 (70.1)	35 (67.3)	66 (75.9)	46 (69.7)	22 (75.9)	28 (59.6)	0.34				
Glycopeptide as the first agents	248 (88.3)	45 (86.5)	80 (92.0)	60 (90.9)	26 (89.7)	37 (78.7)	0.21				
Source control^g^	140/176 (79.6)	21/28 (75.0)	56/62 (93.2)	28/42 (66.7)	14/17 (82.4)	21/27 (77.8)	**0**.**05**	0.93	>0.99	>0.99	>0.99
Infectious disease consultation	191 (68.0)	30 (57.7)	71 (81.6)	39 (59.1)	19 (65.5)	32 (68.1)	**0**.**01**	**0**.**03**	>0.99	>0.99	>0.99
**Outcomes**							** **				
Persistent bacteremia > 7 days	34 (12.1)	2 (3.9)	21 (24.1)	4 (6.1)	2 (6.9)	5 (10.6)	**0**.**002**	**0**.**03**	>0.99	>0.99	>0.99
In-hospital mortality	113 (40.2)	16 (30.8)	49 (56.3)	24 (36.4)	10 (34.5)	14 (29.8)	**0**.**007**	**0**.**03**	>0.99	>0.99	>0.99

Data are presented as no. (%) unless otherwise indicated.

Abbreviations: ST, sequence type; IQR, interquartile range.

^a^ 14 STs are identified in 47 isolates and listed as follows: ST30 (*n* = 14), ST5 (6), ST508 (4), ST338 (3), ST15 (2), ST188 (2), ST398 (2), ST573 (2), ST965 (2), ST3235 (2), ST72 (1), ST89 (1), ST789 (1), ST1232 (1), and non-typable (4).

^b^ Pairwise comparisons with Bonferroni adjustment was performed if overall comparisons were statistically significant (*P*<0.05), and only pairwise comparisons among ST8 vs. different STs were reported. The detailed statistics are shown in Supplementary Table 2. Bold type indicates *P* ≤ 0.05.

^c^ Vascular devices consisted of any type of central vascular catheters, vascular grafts, peripherally inserted central catheters, and any cardiac devices, including heart valves, implantable pacemakers, automated implantable cardioverter-defibrillators, left ventricular assist devices, and extracorporeal membrane oxygenation.

^d^ Device-related infections were defined as infections due to any devices other than vascular device, including prosthetic joints, orthostatic implants, biliary stents, urinary stents, draining tubes, percutaneous feeding tubes, intrathecal catheters, peritoneal dialysis catheters, and oesophageal stents.

^e^ Pleuropulmonary infections included pneumonia, necrotizing pneumonia, lung abscess, and empyema.

^f^ Deep infections were not related to surgery or prosthesis.

^g^ Source control included removal of vascular catheters and foreign devices as well as procedures such as drainage of skin, deep or visceral abscesses, debridement of infected tissue, and operative joint irrigation and drainage.

^h^ Pairwise comparison, *P *< 0.05: ST59 vs. other STs.

^i^ No pairwise comparison showed statistical significance (*P *< 0.05).

^j^ Pairwise comparison, *P *< 0.05: ST239 vs. ST59.

As shown in Table 1, patients with ST8 MRSAB were more likely to have diabetes mellitus (50.0%), and to be from long-term care facilities (25.0%), while they were most unlikely to have a vascular device at the onset of MRSAB (26.9%). A higher proportion of patients with ST8 MRSAB had skin and soft tissue infections (SSTI) (25.0%), endocarditis (7.7%), and deep abscess (7.7%) as the primary focus, despite overall comparisons not meeting statistical significance. Seventy percent of MRSAB patients received effective anti-MRSA therapy within 48 h after onset of bacteraemia, and glycopeptide was the most common drug (88.3%) as the first therapeutic agent without significant difference among different causative STs.

**Figure 2.**
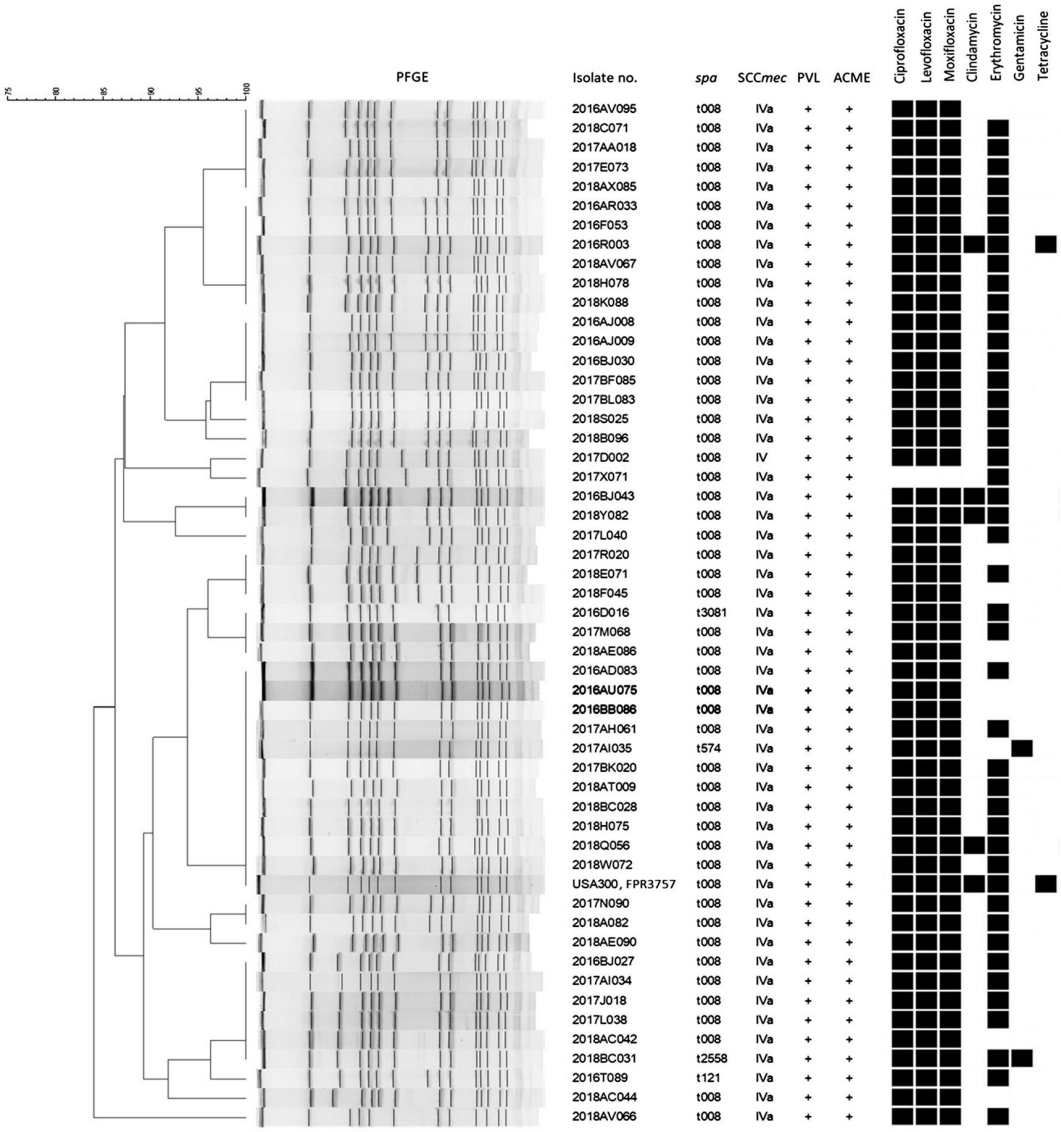
Molecular characteristics of 52 ST8 methicillin-resistant *Staphylococcus aureus* blood isolates. Spa types t121, t574, t2558, and t3081 are variants of t008. Plus symbols indicate detection of a given gene, and black squares indicate resistance to a given antibiotic. Abbreviations are as follows: PFGE, pulsed-field gel electrophoresis; no., number; SCC*mec*, staphylococcal cassette chromosome *mec*; PVL, Pantone-Valentine leucocidin-encoding genes; ACME, arginine catabolic mobile element-encoding genes.

For the 52 ST8 isolates in the present cohort and the USA300 reference strain, FPR3757, the dendrogram via PFGE, SCC*mec* typing, and *spa* typing is shown in Figure 2. The overall similarities were 84.0% by the banding pattern of PFGE. All ST8 isolates carried SCC*mec* IVa, except one as SCC*mec* IV. Using *spa* typing, almost all (*n* = 48) were categorized as t008, and the others each included one of t121, t574, t2558, and t3081, clustered into *spa*-Clonal Complex008 (*spa*-CC008). All the 52 ST8 isolates were positive for both PVL and AMCE (Figure 2), and thus all of them were considered to belong to USA300.

As shown in Table 2, nearly all 281 isolates were susceptible to common parental anti-MRSA agents, including vancomycin, daptomycin and linezolid. Only two ST239 isolates were not susceptible to daptomycin. ST8 isolates were generally susceptible to other non-β-lactam antimicrobial agents, except for being resistant to moxifloxacin and erythromycin (resistance rates, 98.1% and 82.7%, respectively). ST8 isolates were also resistant to ciprofloxacin and levofloxacin (Figure 2), while FPR3757 demonstrated not only resistance to different quinolones and erythromycin, but also showed resistance to clindamycin and tetracycline. ST239 isolates showed its multi-drug resistant (MDR) nature, except for being susceptible to rifampin (94.3%). About half of ST59 isolates were resistant to gentamicin and tetracycline, and nearly all were resistant to clindamycin and erythromycin (Table 2). By pairwise comparisons, ST8 was significantly less susceptible to moxifloxacin compared to ST59, ST45, or other STs (all *P* < 0.001), except ST239.

**Table 2.** Comparisons of in vitro susceptibilities of methicillin-resistant *Staphylococcus aureus* bloodstream isolates among different sequencing types (ST) to key antimicrobial agents tested.

	Total (*n* = 281)	ST8 (*n* = 52)	ST239 (*n* = 87)	ST59 (*n* = 66)	ST45 (*n* = 29)	Other STs^a^ (*n* = 47)	*P*-value^b^				
							Overall	ST8 vs. ST239	ST8 vs. ST59	ST8 vs. ST45	ST8 vs. Other STs
**Vancomycin**											
Range (mg/L)	0.5–2	0.5–1	0.5–2	0.5–1	0.5–1	0.5–1					
MIC_50_/MIC_90_ (mg/L)	1/1	1/1	1/2	1/1	1/1	1/1					
S, n (%)	281 (100.0)	52 (100.0)	87 (100.0)	66 (100.0)	29 (100.0)	47 (100.0)	>0.99				
**Daptomycin**											
Range (mg/L)	0.5–4	0.5–0.5	0.5–4	0.5–0.5	0.5–0.5	0.5–0.5					
MIC_50_/MIC_90_ (mg/L)	0.5/0.5	0.5/0.5	0.5/1	0.5/0.5	0.5/0.5	0.5/0.5					
S, n (%)	279 (99.3)	52 (100.0)	85 (97.7)	66 (100.0)	29 (100.0)	47 (100.0)	0.34				
**Linezolid**											
Range (mg/L)	1–4	2–2	1–2	2–4	2–4	2–4					
MIC_50_/MIC_90_ (mg/L)	2/2	2/2	2/2	2/2	2/2	2/2					
S, n (%)	281 (100.0)	52 (100.0)	87 (100.0)	66 (100.0)	29 (100.0)	47 (100.0)	>0.99				
**Gentamicin**											
Range (mg/L)	2–>16	2–>16	2–>16	2–>16	2–>16	2–>16					
MIC_50_/MIC_90_ (mg/L)	8/>16	2/2	32/>16	2/>16	2/16	2/>16					
S, n (%)	137 (48.8)	50 (96.2)	3 (3.5)	38 (57.6)	16 (55.2)	30 (63.8)	<0.001	<0.001	<0.001	<0.001	<0.001
**Rifampin**											
Range (mg/L)	0.5–>4	0.5–0.5	0.5–>4	0.5–0.5	0.5–0.5	0.5–2					
MIC_50_/MIC_90_ (mg/L)	0.5/0.5	0.5/0.5	0.5/0.5	0.5/0.5	0.5/0.5	0.5/2					
S, n (%)	270 (96.1)	52 (100.0)	82 (94.3)	66 (100.0)	29 (100.0)	41 (87.2)	0.002	0.85	>0.99	>0.99	0.009
**TMP/SMX**											
Range (mg/L)	0.5–>4	0.5–0.5	0.5–>4	0.5–>4	0.5–0.5	0.5–>4					
MIC_50_/MIC_90_ (mg/L)	0.5/>4	0.5/0.5	>4/>4	0.5/0.5	0.5/0.5	0.5/1					
S, n (%)	193 (68.7)	52 (100.0)	4 (4.6)	64 (97.0)	29 (100.0)	44 (93.6)	<0.001	<0.001	>0.99	>0.99	0.73
**Tetracycline**											
Range (mg/L)	2–>16	2–>16	2–>16	2–>16	2–>16	2–>16					
MIC_50_/MIC_90_ (mg/L)	16/>16	2/2	>16/>16	16/>16	>16/>16	2/>16					
S, n (%)	138 (49.1)	51 (98.1)	5 (5.8)	31 (47.0)	13 (44.8)	38 (80.9)	<0.001	<0.001	<0.001	<0.001	0.19
**Moxifloxacin**											
Range (mg/L)	0.25–>4	0.25–>4	0.25–>4	0.25–0.25	0.25–>4	0.25–>4					
MIC_50_/MIC_90_ (mg/L)	2/4	2/2	4/>4	0.25/0.25	2/2	0.25/4					
S, n (%)	118 (42.0)	1 (1.9)	4 (4.6)	66 (100.0)	12 (41.4)	35 (74.5)	<0.001	>0.99	<0.001	<0.001	<0.001
**Clindamycin**											
Range (mg/L)	0.5–>2	0.5–>2	0.5–>2	0.5–>2	0.5–>2	0.5–>2					
MIC_50_/MIC_90_ (mg/L)	>2/>2	0.5/0.5	>2/>2	>2/>2	0.5/0.5	0.5/>2					
S, n (%)^c^	97 (34.5)	48 (92.3)	4 (4.6)	5 (7.6)	17 (58.6)	23 (48.9)	<0.001	<0.001	<0.001	<0.001	<0.001
**Erythromycin**											
Range (mg/L)	0.25–>4	0.25–>4	0.5–>4	0.25–>4	0.25–>4	0.25–>4					
MIC_50_/MIC_90_ (mg/L)	>4/>4	>4/>4	>4/>4	>4/>4	0.5/>4	>4/>4					
S, n (%)^c^	56 (19.9)	9 (17.3)	4 (4.6)	5 (7.6)	17 (58.6)	21 (44.7)					

^a^ Of 47 isolates, 14 STs were identified and listed as follows: ST30 (*n* = 14), ST5 (6), ST508 (4), ST338 (3), ST15 (2), ST188 (2), ST398 (2), ST573 (2), ST965 (2), ST3235 (2), ST72 (1), ST89 (1), ST789 (1), ST1232 (1), and non-typable (4).

^b^ Only pairwise comparisons with Bonferroni adjustment among ST8 vs. different STs were reported if overall comparisons were statistically significant (*P*<0.05).

^c^ For isolates that tested erythromycin resistant and clindamycin susceptible or intermediate, testing for inducible clindamycin resistance by broth microdilution was performed to determine susceptibility to clindamycin.

As shown in Table 1, the overall mortality of patients with MRSAB was 40.2%. Rates of persistent bacteraemia and in-hospital mortality among patients with ST8 MRSAB were significantly lower than those among patients with ST239 MRSAB (3.9% vs. 24.1%, and 30.8% vs. 56.3%, respectively) but equivalent to those among patients with ST59 MRSAB. Predictors associated with in-hospital mortality were further analysed by performing the logistic regression analysis (Table 3). In univariable analysis, HA-MRSAB, a vascular device at onset, shock, the Pitt bacteraemia score, SSTI, pleuropulmonary infection, ST239, and isolates with vancomycin MIC of 1 or 2 mg/L (MIC of 0.5 mg/L as reference) were significant predictors for mortality. In addition to shock (adjusted odds ratio [aOR], 3.16; 95% confidence interval [CI], 1.52–6.60), and the Pitt bacteraemia score (aOR, 1.26; 95% CI, 1.10–1.45), isolates with vancomycin MIC of 1 mg/L (aOR, 2.84; 95% CI, 1.01–7.98) and of 2 mg/L (aOR, 8.45; 95% CI, 2.08–34.38) were significantly associated with mortality in a dose-response manner by multivariable analysis. On the contrary, SSTI (aOR, 0.39; 95% CI, 0.18–0.87) was conversely associated with mortality.

**Table 3.** Univariable and multivariable logistic regression analyses of predictors for in-hospital mortality of patients with methicillin-resistant *Staphylococcus aureus* bacteraemia

Predictors^a^	Death (*n* = 113)	Survival (*n* = 168)	Univariable OR (95% CI)	*P*	Multivariable^b^ OR (95% CI)	*P*
**Demographics**						
Age, y, median (IQR)	67 (58–81)	70 (58–81)	1.00 (0.98–1.01)	0.83		
Gender, male	70 (62.0)	108 (64.3)	0.90 (0.55–1.48)	0.69		
**Onset**						
Community associated	15 (13.3)	38 (22.6)	as reference			
Healthcare-associated, community onset	37 (32.7)	58 (34.5)	1.62 (0.78–3.34)	0.20		
Hospital acquired	61 (54.0)	72 (42.9)	2.15 (1.08–4.27)	0.03		
**Underlying diseases or conditions**						
Cardiovascular diseases	39 (34.5)	54 (32.1)	1.11 (0.67–1.84)	0.68		
Respiratory diseases	25 (22.1)	32 (19.1)	1.21 (0.67–2.17)	0.53		
Neurology diseases	24 (21.2)	27 (16.1)	1.40 (0.76–2.59)	0.27		
Hepatobiliary diseases	26 (23.0)	31 (18.5)	1.32 (0.73–2.37)	0.35		
Chronic renal impairment	33 (29.2)	58 (34.5)	0.78 (0.47–1.31)	0.35		
Rheumatology diseases	10 (8.9)	11 (6.6)	1.39 (0.57–3.38)	0.69		
Diabetes mellitus	31 (27.4)	61 (36.3)	0.66 (0.39–1.11)	0.12		
Solid tumour	48 (42.5)	62 (36.9)	1.26 (0.78–2.06)	0.35		
Haematological malignancy	6 (5.3)	8 (4.8)	1.12 (0.38–3.32)	0.84		
Charlson comorbidity index, median (IQR)	4 (3–6)	4 (2–6)	1.03 (0.94–1.13)	0.52		
Chronic dialysis	13 (11.5)	31 (18.5)	0.57 (0.29–1.15)	0.12		
Long-term care facility	13 (11.5)	20 (11.9)	0.96 (0.46–2.02)	0.92		
Vascular device at onset	66 (58.4)	71 (42.3)	1.92 (1.18–3.11)	0.008		
**Severity**						
Shock	53 (46.9)	22 (13.1)	5.86 (3.28–10.48)	<0.001	3.16 (1.52–6.60)	0.002
Pitt bacteraemia score, median (IQR)	3 (1–5)	1 (0–2)	1.43 (1.27–1.62)	<0.001	1.26 (1.10–1.45)	0.001
**Infection focus**						
Primary	43 (38.1)	62 (36.9)	1.05 (0.64–1.72)	0.85		
Catheter-related	29 (25.7)	42 (25.0)	1.04 (0.60–1.79)	0.90		
Device-related	3 (2.7)	7 (4.2)	0.63 (0.16–2.48)	0.51		
Skin and soft tissue	13 (11.5)	36 (21.4)	0.48 (0.24–0.95)	0.03	0.39 (0.18–0.87)	0.02
Pleuropulmonary	22 (19.5)	17 (10.1)	2.15 (1.08–4.26)	0.03		
Native osteoarticular	1 (0.9)	6 (3.6)	0.24 (0.03–2.03)	0.19		
Endocarditis	8 (7.1)	6 (3.6)	2.06 (0.69–6.10)	0.19		
Septic thrombophlebitis	2 (1.8)	4 (2.4)	0.74 (0.13–4.10)	0.73		
Deep abscess	2 (1.8)	8 (4.8)	0.36 (0.08–1.73)	0.20		
**Management**						
Effective antibiotics within 48 h after onset	77 (68.1)	120 (71.4)	0.86 (0.51–1.44)	0.56		
Glycopeptide as the first agents	96 (85.0)	152 (90.5)	0.59 (0.29–1.23)	0.16		
Source control	60 (82.7)	80 (75.5)	1.95 (0.87–4.35)	0.10		
ID consultation	82 (72.6)	109 (64.9)	1.43 (0.85–2.40)	0.18		
**Sequence types**						
ST8	16 (14.2)	36 (21.4)	as reference			
ST239	49 (43.4)	38 (22.6)	2.90 (1.40–5.99)	0.004		
ST59	24 (21.2)	42 (25.0)	1.29 (0.59–2.79)	0.52		
ST45	10 (8.9)	19 (11.3)	1.18 (0.45–3.11)	0.73		
Other STs	14 (12.4)	33 (19.6)	0.95 (0.40–2.25)	0.92		
**Vancomycin MIC**						
0.5 mg/L	6 (5.3)	22 (13.1)	As reference			
1 mg/L	91 (80.5)	139 (82.7)	2.40 (0.94–6.15)	0.07	2.84 (1.01–7.98)	0.05
2 mg/L	16 (14.2)	7 (4.2)	8.38 (2.36–29.74)	0.001	8.45 (2.08–34.38)	0.003

Data are presented as no. (%) unless otherwise indicated.

Abbreviations: IQR, interquartile range; OR, odds ratios; CI, confidence interval.

^a^ Mann–Whitney *U* test was used to compare continuous variables, and χ^2^ or Fisher exact test was used to compare categorical variables.

^b^ Hosmer and Lemeshow goodness-of-fit test *P* = 0.2292 > 0.05 (df = 9).

## Discussion

In Taiwan, ST59 and ST239 were long recognized as the dominant CA-MRSA and HA-MRSA clones, respectively, until this study [5,6,16]. Surprisingly, ST8 was not uncommon since 2016 but abruptly saw a 1.5-fold increase to become the most prevalent ST in 2018. Further, all ST8 isolates in this report were clustered to USA300 by a consensus definition [7,8]. Our finding that ST8/USA300 was most dominant in CA-MRSAB, followed by HACO-MRSAB and HA-MRSAB, mirrored the dissemination direction of USA300 in the USA and other USA300 endemic regions, suggesting clonal expansion from community to hospital [5,6]. As for the infection focus of MRSAB, ST8/USA300 had the highest proportions for SSTI, endocarditis and deep-seated infections among different STs. Meanwhile, ST8/USA300 generally exhibited susceptibilities to non-β-lactams other than fluoroquinolones.

In parallel, Peng et al. reported ST8 became the second most common clone, causing cellulitis and osteomyelitis in both community and hospital settings from another hospital in Taiwan between 2016 and 2018 [15], while colonization rates of ST8 MRSA varied in Taiwan. Our recent surveillance study targeting healthy adults between 2017 and 2018 [22] demonstrated that the carriage rate of ST8 in Taiwan remained low (<1%), as well as one large-scale longitudinal study from 1995 to 2015, confirming ST8 belonged to USA300 by whole-genome sequencing (WGS) [14]. Another surveillance study, however, showed the carriage rate of ST8 was common (11.3%) in long-term care facilities in 2016 [13]. Whether these patterns simply represent local secular trends or different phases of clonal expansion and/or contraction is unclear [23]. Our finding in this report with others may suggest ST8/USA300 is expected to achieve dominance causing MRSA colonization and infections despite the pre-existence of diverse MRSA genetic background, such as ST59, in the community. Although ST8/USA300 is most prevalent in North/South America [5], its dominance in the rest of the world is to a lesser extent. This is the first report, to our best knowledge, demonstrating ST8/USA300 successfully replaces a prior long-lasting endemic CA-MRSA clone, ST59 in Taiwan, outside the USA.

The reasons for clonal replacement are complex in CA-MRSA evolutions. PVL, SCC*mec* IV, and ACME genes are unique to CA-MRSA in different genetic lineages, which may offer evolutional fitness compared to other MRSA clones [5,23]. In Taiwan, ST59 is positive for PVL with either SCC*mec* IV or SCC*mec* V_T_, mostly without ACME [15], showing similar virulence to USA300 through Agr, α-toxin, and PSMα [24]. Compared to the virulent genetic components of ST59, all ST8/USA300 isolates in the current study had ACME, indicating the role of ACME as the major fitness determinant associated with the current clonal replacement in Taiwan.

Alternatively, we also speculated the increased consumption of fluoroquinolone (FQ) as one of the driving forces to replace FQ sensitive MRSA clones (ST59) with FQ resistant clones (ST8/USA300). Taiwan’s national health insurance (NHI) database showed a reduction in FQ consumptions since 2008 due to antibiotic restrictions in Taiwan [25]. However, a recent study by the National Health Research Institutes in Taiwan showed private drug consumption accounted for a certain amount in the community not counted in the NHI database, likely resulting in underestimation of the true FQ consumption amount [25]. Therefore, the FQ positive selective pressure might remain significant, which in turn facilitated the clonal shift from ST59 to ST8/USA300 in Taiwan.

In addition to the overall similarities of 52 ST8/USA300 isolates by PFGE above 80.0%, our study, using other molecular typing methods, illustrated microevolutions of ST8/USA300 MRSA have developed in Taiwan, as *spa* typing showed four isolates possessed *spa*-CC008, instead of *spa*-t008. One prior study by performing a whole-genome sequence (WGS) to demonstrate the existence of ST8 with local transmission in Taiwan since 2005 further supports our findings [14]. Additionally, the antibiotic resistance patterns of ST8/USA300 isolates in our present study were not totally the same as the USA300 reference strain, FPR3757. Read *et al* also reported changes in plasmid and prophage content and antibiotic resistance of ST8/USA300 MRSA between index infection isolates and subsequent colonization isolates within a person [26]. Both results may reflect a loss of some mobile genetic elements encoding antibiotic resistance during evolutions of ST8/USA300 MRSA.

The aforementioned findings, however, cannot preclude the possibilities of multiple introductions of ST8/USA300 causing the current epidemic in Taiwan, like dissemination of USA300 to other continents [27]. Alternatively, ST8 MSSA may coincidentally acquire SCC*mec* IVa or IV to become ST8 MRSA, then disseminate from community to hospital, but this is less likely, because previous studies have shown extremely low prevalence (<1%) of ST8 MSSAB in Taiwan [28,29]. Further epidemiological studies using WGS analysis are warranted to depict the evolutions and dissemination of ST8/USA300 MRSA from colonization to invasive infections in Taiwan over time.

The role of USA300 in the mortality of patients with MRSAB remains debatable, albeit animal data support increased virulence with USA300 [30]. In the earlier U.S. study, patients with USA300 MRSAB were associated with younger age and more SSTI, and were, in turn, associated with lower mortalities than those infected by USA100 [31]. Later, after intermixing of USA300 and USA100, mortality did not significantly differ between these two clones [32] or become even higher in USA300 [33]. Our data suggests the current situation of MRSAB in Taiwan was more similar to the context of the initial report of USA300 in the USA; thus, the impact of MRSA genotypes on clinical outcomes might be confounded by patient ages and clinical syndromes. Further, our results echo prior results showing vancomycin MIC as a detrimental factor for in-hospital mortality in a dose-response manner with most patients having received glycopeptide as the first anti-MRSA agent in the treatment of MRSAB [34].

Nevertheless, several limitations should be noted in our study. First, this was a single-centre retrospective study. Our study was conducted in a relatively short period; thus, bias was inevitable and generalization should be cautious. However, the Taiwan Surveillance of Antimicrobial Resistance (TSAR) program, conducted biennially to collect different micro-organisms from medical centres and regional hospitals throughout Taiwan since 1998, also found the rates of ST8/USA300 increased from 25.0% in 2016 to 30.0% in 2018 paralleling with the declining rates of ST239 and ST59 among difference clinical specimens, including blood, pus/abscess, and respiratory specimens [35]. Other reports in Taiwan also found ST8/USA300 becoming common in superficial and deep MRSA infections in the same period [15]. All above findings shed light on the emergence of ST8/USA300 as a dominant MRSA clone to cause different clinical syndromes in Taiwan. Conversely, our data lacked several confounding factors, including a history of prior MRSA colonization, household clusters, or prior antibiotic exposure. All warrant further investigation to depict the evolution and dissemination of the current ST8/USA300 epidemic in Taiwan.

Collectively, our study found there was a clonal shift of MRSA from ST239 and ST59 to ST8/USA300, especially in community settings. The long-term trend of MRSA infections in the USA has illustrated ST8/USA300 is difficult to eradicate in the community, therefore, an important task for current MRSA research is to delineate effective strategies to prevent dissemination and colonization of ST8/USA300 MRSA in the community, and subsequently to reduce the burden of MRSA infections. Also, continuing surveillance of molecular epidemiology of MRSA in Asia, especially in Taiwan, is indicated to ascertain whether ST8/USA300 will successfully expand with long-term fitness and to trace its pathogenicity on patient outcomes.

## Supplementary Material

Supplementary_tables_clean_file.docx
