# Supplementary material for: Sequence type 8 as an emerging clone of methicillin-resistant Staphylococcus aureus causing bloodstream infections in Taiwan
Source: Emerg Microbes Infect. 2021 Sep 24;10(1):1908–18. doi: 10.1080/22221751.2021.1981158 (PMC8475108; doi:10.1080/22221751.2021.1981158)
Supplement: Supplementary_tables_clean_file.docx [file TEMI_A_1981158_SM7403.docx]

**Supplementary Table 1.** Distributions of sequencing types (ST) of methicillin-resistant *Staphylococcus aureus* bloodstream isolates

|  | **2016** | **2017** | **2018** | **Overall *P*-value^b^** |
| --- | --- | --- | --- | --- |
|  |  |  |  |  |
| **ST8** | 14 (15.2) | 17 (15.9) | 21 (25.6) | 0.605 |
| **ST239** | 32 (34.8) | 35 (32.7) | 20 (24.4) |  |
| **ST59** | 21 (22.8) | 28 (26.2) | 17 (20.7) |  |
| **ST45** | 11 (12.0) | 10 (9.4) | 8 (9.8) |  |
| **Others ST^a^** | 14 (15.2) | 17 (15.9) | 16 (19.5) |  |
| **Total** | 92 (100) | 107 (100) | 82 (100) |  |

Data are presented as no. (%).

^a^ Of 47 isolates, 14 STs were identified and listed as follows: ST30 (n=14), ST5 (6), ST508 (4), ST338 (3), ST15 (2), ST188 (2), ST398 (2), ST573 (2), ST965 (2), ST3235 (2), ST72 (1), ST89 (1), ST789 (1), ST1232 (1), and non-typable (4).

^b^ Over comparisons was performed by using R by C Chi-square test.

**Supplementary Table 2.** Statistics for comparisons of clinical characteristics and outcomes of 281 patients with methicillin-resistant *Staphylococcus aureus* bacteraemia by sequencing types.

|  | ***P-*value^a^** | | | | | | | | | | | |
| --- | --- | --- | --- | --- | --- | --- | --- | --- | --- | --- | --- | --- |
|  | **Overall** | **ST8 vs. ST239** | **ST8 vs. ST59** | **ST8 vs. ST45** | **ST8 vs. Other STs**^b^ | **ST239 vs. ST59** | | **ST239 vs. ST45** | **ST239 vs. Other STs**^b^ | **ST59 vs. ST45** | **ST59 vs. Other STs**^b^ | **ST45 vs. Other STs**^b^ |
| **Demographics** |  |  |  |  |  |  |  | |  |  |  |  |
| Age in year | 0.09 |  |  |  |  |  |  | |  |  |  |  |
| Gender, Male | 0.30 |  |  |  |  |  |  | |  |  |  |  |
| **Underlying diseases or conditions** |  |  |  |  |  |  |  | |  |  |  |  |
| Cardiovascular diseases | 0.41 |  |  |  |  |  |  | |  |  |  |  |
| Respiratory diseases | 0.17 |  |  |  |  |  |  | |  |  |  |  |
| Neurology diseases | 0.07 |  |  |  |  |  |  | |  |  |  |  |
| Hepatobiliary diseases | 0.51 |  |  |  |  |  |  | |  |  |  |  |
| Chronic renal impairment | 0.99 |  |  |  |  |  |  | |  |  |  |  |
| Chronic dialysis | 0.99 |  |  |  |  |  |  | |  |  |  |  |
| Rheumatology diseases | 0.90 |  |  |  |  |  |  | |  |  |  |  |
| Diabetes mellitus | **0.05** | **0.04** | 0.09 | 0.80 | 0.90 | >0.99 | >0.99 | | >0.99 | >0.99 | >0.99 | >0.99 |
| Solid tumour | **0.003** | >0.99 | 0.08 | 0.29 | >0.99 | 0.11 | 0.45 | | >0.99 | >0.99 | **0.02** | 0.09 |
| Haematological malignancy | 0.07 |  |  |  |  |  |  | |  |  |  |  |
| Charlson comorbidity index | 0.32 |  |  |  |  |  |  | |  |  |  |  |
| Long-term care facility | **0.02** | 0.09 | **0.02** | >0.99 | **0.04** | >0.99 | >0.99 | | >0.99 | >0.99 | >0.99 | >0.99 |
| Vascular device at onset^c^ | **<0.001** | **<0.001** | 0.38 | 0.55 | >0.99 | **0.02** | 0.34 | | **0.003** | >0.99 | >0.99 | >0.99 |
| **Severity** |  |  |  |  |  |  |  | |  |  |  |  |
| Shock | **0.04** | >0.99 | >0.99 | >0.99 | >0.99 | **0.06** | 0.68 | | 0.19 | >0.99 | >0.99 | >0.99 |
| Pitt bacteraemia score | **0.02** | 0.84 | >0.99 | >0.99 | >0.99 | **0.02** | 0.39 | | 0.35 | >0.99 | >0.99 | >0.99 |
| **Infection focus** |  |  |  |  |  |  |  | |  |  |  |  |
| Primary | 0.26 |  |  |  |  |  |  | |  |  |  |  |
| Catheter-related | **0.003** | **0.003** | 0.98 | 0.12 | >0.99 | 0.44 | >0.99 | | 0.23 | >0.99 | >0.99 | >0.99 |
| Device-related^d^ | 0.21 |  |  |  |  |  |  | |  |  |  |  |
| Skin and soft tissue | 0.17 |  |  |  |  |  |  | |  |  |  |  |
| Pleuropulmonary^e^ | 0.23 |  |  |  |  |  |  | |  |  |  |  |
| Native osteoarticular | 0.64 |  |  |  |  |  |  | |  |  |  |  |
| Endocarditis | 0.82 |  |  |  |  |  |  | |  |  |  |  |
| Septic thrombophlebitis | 0.85 |  |  |  |  |  |  | |  |  |  |  |
| Deep infection^f^ | 0.17 |  |  |  |  |  |  | |  |  |  |  |
| **Management** |  |  |  |  |  |  |  | |  |  |  |  |
| Effective antibiotics within 48 hours after onset | 0.34 |  |  |  |  |  |  | |  |  |  |  |
| Glycopeptide as the first agents | 0.21 |  |  |  |  |  |  | |  |  |  |  |
| Source control^g^ | **0.05** | 0.93 | >0.99 | >0.99 | >0.99 | **0.03** | >0.99 | | >0.99 | >0.99 | >0.99 | >0.99 |
| Infectious disease consultation | **0.01** | **0.03** | >0.99 | >0.99 | >0.99 | **0.03** | >0.99 | | >0.99 | >0.99 | >0.99 | >0.99 |
| **Outcomes** |  |  |  |  |  |  |  | |  |  |  |  |
| Persistent bacteraemia > 7 days | **0.002** | **0.03** | >0.99 | >0.99 | >0.99 | **0.006** | 0.12 | | 0.20 | >0.99 | >0.99 | >0.99 |
| In-hospital mortality | **0.007** | **0.03** | >0.99 | >0.99 | >0.99 | 0.12 | 0.36 | | **0.03** | >0.99 | >0.99 | >0.99 |

^a^ Pairwise comparisons with Bonferroni adjustment was performed if overall comparisons were statistically significant (*P*<0.05), which are shown in bold type. ^b^ 14 STs are identified in 47 isolates and listed as follows: ST30 (n=14), ST5 (6), ST508 (4), ST338 (3), ST15 (2), ST188 (2), ST398 (2), ST573 (2), ST965 (2), ST3235 (2), ST72 (1), ST89 (1), ST789 (1), ST1232 (1), and non-typable (4).

^c^ Vascular devices consisted of any type of central vascular catheters, vascular grafts, peripherally inserted central catheters, and any cardiac devices, including heart valves, implantable pacemakers, automated implantable cardioverter-defibrillators, left ventricular assist devices, and extracorporeal membrane oxygenation.

^d^ Device-related infections were defined as infections due to any devices other than vascular device, including prosthetic joints, orthostatic implants, biliary stents, urinary stents, draining tubes, percutaneous feeding tubes, intrathecal catheters, peritoneal dialysis catheters, and oesophageal stents.

^e^ Pleuropulmonary infections included pneumonia, necrotizing pneumonia, lung abscess, and empyema.

^f^ Deep infections were not related to surgery or prosthesis.

^g^ Source control included removal of vascular catheters and foreign devices as well as procedures such as drainage of skin, deep or visceral abscesses, debridement of infected tissue, and operative joint irrigation and drainage.
